# Look at me now! Enfacement illusion over computer-generated faces

**DOI:** 10.3389/fnhum.2023.1026196

**Published:** 2023-03-09

**Authors:** Stefania La Rocca, Silvia Gobbo, Giorgia Tosi, Elisa Fiora, Roberta Daini

**Affiliations:** ^1^Department of Psychology, University of Milano–Bicocca, Milan, Italy; ^2^MiBTec–Mind and Behavior Technological Center, University of Milano–Bicocca, Milan, Italy; ^3^Department of History, Society and Human Studies, University of Salento, Lecce, Italy

**Keywords:** enfacement, face processing, computer-generated faces, embodiment, visual perception

## Abstract

According to embodied cognition research, one’s bodily self-perception can be illusory and temporarily shifted toward an external body. Similarly, the so-called “enfacement illusion” induced with a synchronous multisensory stimulation over the self-face and an external face can result in implicit and explicit changes in the bodily self. The present study aimed to verify (i) the possibility of eliciting an enfacement illusion over computer-generated faces and (ii) which multisensory stimulation condition was more effective. A total of 23 participants were asked to look at a gender-matched avatar in three synchronous experimental conditions and three asynchronous control conditions (one for each stimulation: visuotactile, visuomotor, and simple exposure). After each condition, participants were asked to complete a questionnaire assessing both the embodiment and the enfacement sensations to address different facets of the illusion. Results suggest a stronger effect of synchronous vs. asynchronous stimulation, and the difference was more pronounced for the embodiment items of the questionnaire. We also found a greater effect of visuotactile and visuomotor stimulations as compared to the simple exposure condition. These findings support the enfacement illusion as a new paradigm to investigate the ownership of different face identities and the specific role of visuotactile and visuomotor stimulations with virtual reality stimuli.

## 1. Introduction

The self-face is recognized as a special stimulus for our face-processing system, as shown by both behavioral and neuroimaging studies (Devue and Brédart, 2011; Bortolon and Raffard, 2018; Alzueta et al., 2020). Notably, an advantage of one’s face has been observed in face processing (i.e., self-face advantage; Sugiura et al., 2005; Ma and Han, 2010). This effect has been documented even in participants with difficulties recognizing faces, namely congenital prosopagnosics (Malaspina et al., 2018).

Thus, causing participants to identify with presented faces could, in principle extend the self-face advantage to faces other than one’s own. This can lead to a new range of possibilities in the domain of face processing research.

A temporary change in one’s self-representation can be induced by balancing multisensory information (Tsakiris, 2010). This change has been observed both with bodies (Lenggenhager et al., 2007) and faces (Tsakiris, 2008). The experimental procedures addressing the body are named body illusions and refer to the embodiment effect, which is the experience of ownership over a fake body (or parts of it). Crucially, when faces are concerned, this effect is called “Enfacement illusion”(Sforza et al., 2010).

Different methods have been proposed to induce the enfacement illusion, namely visuotactile, visuomotor, and visuotactile-motor stimulations (Porciello et al., 2018). In the visuotactile stimulation, participants look at another face in front of them while both their face and the other one are touched by a stick (Tsakiris, 2008; Sforza et al., 2010; Tajadura-Jiménez et al., 2012a,b). The touch can be synchronous with the viewed face in terms of timing and location (synchronous condition), or asynchronous (asynchronous condition). The latter usually serves as a control condition because it seems not to induce the illusion (Porciello et al., 2018). In particular, studies suggest that temporal synchrony is more important than spatial synchrony in inducing the effect (Apps et al., 2015). The visuomotor stimulation consists of participants viewing a face in front of them while being instructed to produce head movements. Movements can be synchronized with the viewed face (synchronous condition) or not (asynchronous condition). Again, the asynchronous condition usually serves as a control (Serino et al., 2015). Active movements (i.e., movements that are controlled by the participant) are suggested to be more effective than passive movements (i.e., manipulated by the experimenter) in eliciting the embodiment effect in a classical rubber hand illusion paradigm (Dummer et al., 2009). However, the effect observed by Dummer et al. (2009) was only marginally significant; moreover, another study did not find significant differences between active and passive movements (Kalckert and Ehrsson, 2014). Therefore, it is not completely clear whether movement needs to be active to elicit embodiment or enfacement. The third type of stimulation described in the literature concerns visuotactile-motor stimulation: participants perceive a touch resulting from a movement generated by themselves (Tajadura-Jiménez et al., 2013), which can be synchronous or asynchronous with the observed face. To the best of our knowledge, in enfacement paradigms the difference between active and passive stimulation in pure visuomotor condition has not been investigated. As described in Dummer et al. (2009) for embodiment, active stimulation refers to a movement elicited by the participant while passive stimulation is elicited by the experimenter. However, for enfacement illusion, there is a third possibility, that has never been studied, in which the participant moves the head actively by following the video, without an online pairing of the avatar and participants movements. In this way the participants do not have a real control over the avatar movements, but there is only an illusory control. Therefore, we decided to refer to this possibility in enfacement paradigm as guided movement. It is worth considering that even mere exposure to a body part can elicit embodiment (Dasgupta and Rivera, 2008; La Rocca et al., 2020). To our knowledge, the exposure condition has not yet been investigated with faces.

The literature reports different enfacement illusion paradigms. Some authors used two people sitting in front of each other (Sforza et al., 2010; Bufalari et al., 2019) others used movies displaying real unfamiliar faces (Tsakiris, 2008; Tajadura-Jiménez et al., 2012b; Panagiotopoulou et al., 2017; Gülbetekin et al., 2021) or humanoid animated characters (Gonzalez-Franco et al., 2020). More recently, the enfacement literature introduced the use of the 3-D personalized reconstruction of faces (Grewe et al., 2021) and other standardized avatars (Serino et al., 2015).

Using computer-generated (CG) faces has become increasingly common in different psychological research areas (Yaros et al., 2019). Artificial faces with a very human-like appearance can now be generated by several software programs (either “from scratch” or by inputting real photographs to be converted into 3-D head models). Once generated, the faces can then be manipulated for perceptual or psychological characteristics (e.g., expressions, viewpoint, emotions, and feature size).

Computer-generated faces can differ according to their human likeness, which describes the degree to which an entity has a human-like appearance and presents human physical traits. Furthermore, they can present different levels of photographic realism and physical appearance details (e.g., rendering, shades, and texture). The most notable difference between these CG faces and face photographs is that the CG faces appear to lack fine-grained surface texture information and imperfections that are usually present in photographic face stimuli. New recent software has been developed allowing the creation of highly realistic 3-D faces starting with face photographs (i.e., Character Creator).^1^ To the best of our knowledge, no research in psychology has used this program to study face perception. Being able to edit the avatar’s characteristics could influence the embodiment illusion experience (Tsakiris and Haggard, 2005; Lugrin et al., 2015) together with its perception and attitudes toward it (Peck et al., 2013).

The advantages of this type of stimuli is to expose participants to faces that are as realistic as possible and at the same time are editable under a variety of aspects (e.g., facial expressions, facial features, gender, and social cognition manipulation).

The present study aimed to (i) test the possibility of eliciting the enfacement illusion over virtual faces and (ii) verify which enfacement paradigm elicits a stronger illusion. Particularly, we aimed to verify whether simple exposure to faces without multisensory integration is sufficient in eliciting enfacement or if a multisensory and congruent stimulation is necessary. To do so, (i) Computer Generated faces were created through the software Character Creator and (ii) we compared enfacement and embodiment illusions in visuotactile stimulation, visuomotor stimulation, and exposure condition.

Having a methodological reference for studying enfacement through the use of avatars can lead to a wide range of applications in virtual reality experiments both in the cognitive and social domains.

## 2. Methods

### 2.1. Participants

A total of 24 young adults participants took part in the study. All participants were caucasian and we excluded those wearing glasses and having beard, in order to have an homogenous group. The number of participants was calculated *a priori* through the software G*power 3.1.9.4. We referred to the recent literature about embodiment phenomena in VR (La Rocca et al., 2020; Tosi et al., 2020, 2021) that suggests a medium effect size for the experimental condition (eta-squared around 0.13). We run an *a priori* power analysis for a within-subjects repeated measure ANOVA encompassing a 2 × 3 × 2 design. The analysis revealed that to reach a power of 0.80, with alpha set to 0.05 and effect size set to 0.30, 24 participants were needed. The final number of participants is 23 [13 females, mean age = 25.47 (SD = 2.76), age range = 21–34], as the first participant was removed from the analyses due to a technical error. The reported research protocol was approved by the ethical committee of the University of Milano-Bicocca (protocol number: RM-2021-392), and written informed consent was obtained from all participants.

### 2.2. Stimuli

We generated four avatars, two males and two females, which had been shown, respectively to male and female participants. Avatars were created starting from two pictures belonging to the Chicago Face Database (Ma et al., 2015) having a suitability score above 4. Subsequently, they were morphed through the program Character Creator 3. Each photo underwent a digital transformation with the “Headshot” Plug-In. This plug-in can edit faces *via* pro-mode and auto-mode. The first one is designed for high-resolution texture processing and facial morph definition. Auto-mode makes a lower definition avatar but allows to generate 3-D hair starting from the original photo. We processed *via* auto-mode to generate 3-D hair and then converted our stimuli through pro-mode to obtain highly realistic faces.

**FIGURE 1:**
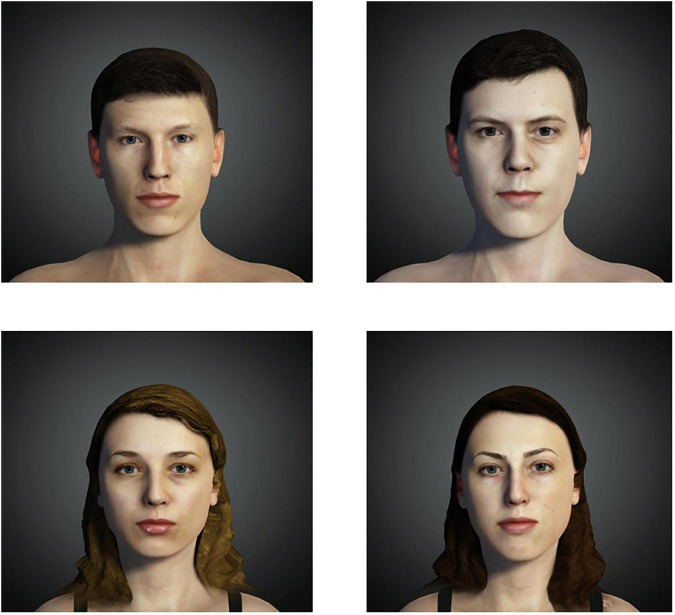
Males and females pictures of the used avatars.

Once the avatars were created, each one was inserted in an environment resembling the lab used for testing, edited through the “iClone 3DXChange 7” pipeline, and converted to.avi format videos. Videos were created to belong to one of the three experimental conditions: visuotactile stimulation, visuomotor stimulation, and simple exposure. Moreover, the respective control condition videos were created. Figure 1 shows the avatars.

### 2.3. Procedure

Each participant underwent six conditions (experimental: synchronous visuotactile, synchronous visuomotor, synchronous exposure; control: asynchronous visuotactile, asynchronous visuomotor, asynchronous exposure). The experimental setting matched the lab environment re-created in the videos. Participants sat in front of a screen and, with the back leaned, the distance of the eyes from the monitor was approximately 50 cm. The screen height with respect to the floor was adjusted to resemble a mirror by aligning it with the participant’s head.

The six conditions were administered in a counterbalanced order: we created 24 unique combinations of order presentation, and each one was presented to one of our participants. Each participant was exposed to one of the avatar matching his/her gender, whose identity was assigned in a counterbalanced order.

In the case of the synchronous visuotactile condition, the video showed an avatar that stayed still while a chopstick touched his/her cheek at a frequency of 1 Hz (the pace was given by the metronome) for 2 min. To ensure that the touch was realistic, the cheek was edited to reproduce the skin reaction to a touch in that position. While viewing the avatar being touched on his/her cheek by the chopstick, participants received a synchronous tactile stimulation by the experimenter. Touches were delivered on the corresponding location of the participant’s cheek at a frequency of 1 Hz following the same pace given by the metronome in the video. In the asynchronous control visuotactile condition, the chopstick touched the avatar’s cheek in random same positions and with an anti-phasic rhythm. During the video, the experimenter touched the participants’ cheek with the same rhythm as in the synchronous condition. However, the effective touch did not match the video either with respect to the location or the rhythm of the observed touch. The metronome was still active to maintain equal circumstances.

In the case of the synchronous visuomotor condition, the video showed an avatar that was modified to produce either a nodding or a shaking guided movement. Half of the participants were presented with the nodding movement, the other half saw the shaking one. The movement was regular and followed the rhythm of a head movement per second. Participants were instructed to nod/shake their heads following the same pace given by the metronome in the video. In the asynchronous visuomotor condition, the avatar produced the movement (nodding or shaking) following a random rhythm. Participants received the same instructions as in the synchronous one, but the observed avatar did not match their movement (see Section “2.2. Stimuli”). Participants were instructed to move following the metronome. This served to create the illusion of controlling the avatar’s movements in the synchronous condition. On the other hand, in the asynchronous condition it served to de-synchronize participants’ and avatars’ movements. However, there was not registration of participants’ actual movements through face expressions and movements online trackers. The movements were externally guided.

In the congruent exposure condition, the avatar was presented as static and in the same position as the participants’ faces. In the control incongruent exposure condition, the avatar was presented as static and inverted. During both the synchronous and control exposure conditions, participants were only instructed to look at the avatar for 2 min. Each video lasted 2 min.

In order to make the results comprehensible and comparable to the other condition, we will refer to the congruent and incongruent exposure conditions as synchronous and asynchronous.

**FIGURE 2:**
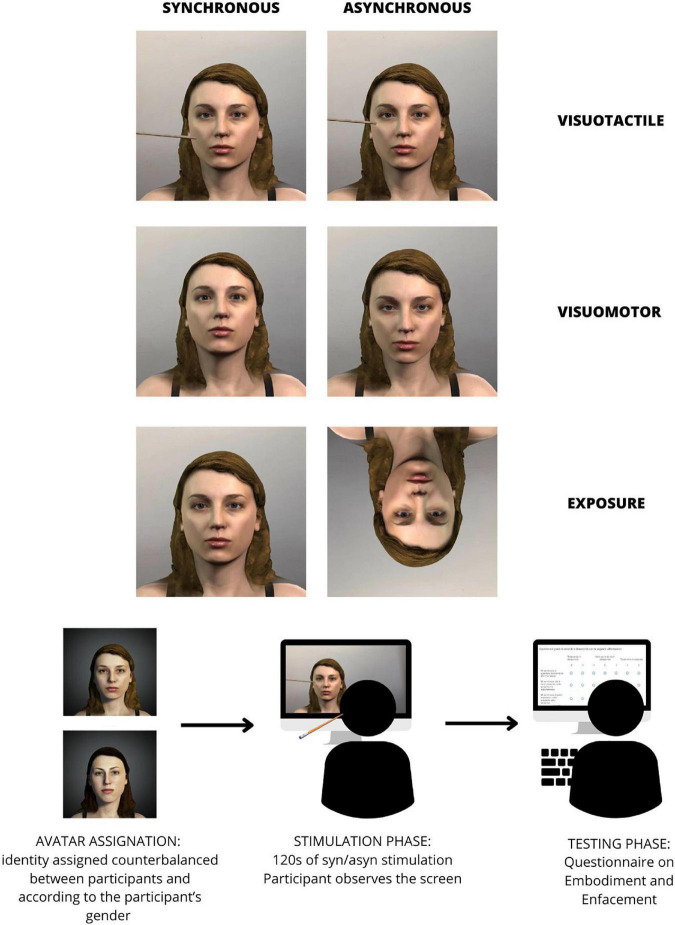
Description of the procedure of the experiment. Enfacement was elicited through three conditions, each in a synchronous (congruent) or asynchronous (incongruent) modality. Conditions were visuo-tactile stimulation, guided movement visuo-motor stimulation or simple exposure. Each participant was assigned one of two avatars in counterbalanced order to match his/her gender. After being exposed to each condition in each modality, participants completed the questionnaires about enfacement. Also stimulations were administered in counterbalanced order.

After each condition, participants answered 16 self-report questions to assess their subjective experience during the video (a schematic representation of the procedure can be found in Figure 2). The first six questions (Q1–Q6) belonged to a questionnaire used for investigating the embodiment effect (Tosi et al., 2020, 2021; Tosi and Romano, 2022). Items were re-adapted to be specific for face stimuli. The following ten questions (Q7–Q16) belonged to the enfacement questionnaire (Tajadura-Jiménez et al., 2012b). We removed eight questions from the original enfacement questionnaire as they were specifically related to the visuotactile condition (i.e., questions 1 and 2) or to the visuomotor condition (i.e., question 8) or unrelatable according to our experimental paradigm (i.e., questions 11, 12, 15, 17, 18). The complete list of the questions is reported in Table 1.

**TABLE 1:** Items of the embodiment and enfacement questionnaires.

Subcomponents		ID	Question
Embodiment	Ownership	Q1	It seems like I was looking directly at my own face
		Q2	It seems like the face in the video belonged to me
	Agency	Q3	It seems like I could have moved the face in the video?
		Q4	It seems like I was not in control of the face in the video?
	Location	Q5	It seems like the face in the video was in the location where my face was
		Q6	It seems like I could have felt a touch given to the face in the video
Enfacement		Q7	I felt like the other’s face was my face
		Q8	It seemed like the other’s face belonged to me
		Q9	It seed like I was looking at my own mirror reflection
		Q10	It seemed like the other’s face began to resemble my own face
		Q11	It seemed like my own face began to resemble the other person’s face
		Q12	It seemed like my own face was out of my control
		Q13	It seemed like the experience of my face was less vivid than normal
		Q14	It seemed like the person in the video was attractive
		Q15	It seemed like the person in the video was trustworthy
		Q16	I felt that the other person was imitating me

It might be noted that items are translated from Italian to English for publication purposes. However, Q5 might sounds unclear, but it was adapted referring to the position of the face and not its location in space.

Participants were asked to indicate their level of agreement with each question on a seven-point Likert scale (from–3—disagreement–to + 3—agreement). Before the analyses, we reversed the item Q4.

Participants were also administered the self-esteem IAT, as part of a wider project. However, the results will not be discussed in the present article.

The overall experimental design consisted of a 2 (Congruency) × 3 (Stimulation) × 2 (Questionnaire) within-subject design. The following dimensions were assessed: Congruency (i.e., synchronous experimental condition vs. asynchronous control condition), Stimulation (i.e., visuotactile, visuomotor, and exposition), and Questionnaire (embodiment items vs. enfacement items). The videos of the experimental conditions and the dataset are available on the Open Science Framework platform at the following link: https://osf.io/cf8qv/?view_only=efd1bb4b124a4c12b295c5f31ea8bc20.

### 2.4. Analyses

Before running the analyses, each participant’s responses to the questionnaire have been ipsatized by centering the responses on the average score of all the questions in all the conditions and dividing the resulting value by the standard deviation of the whole set of responses. The procedure is a within-subject normalization and removes the response set bias (i.e., the participant’s response style). Thus, each item is coded in terms of standard deviations from each participant’s average response (Hofstede, 1984). We then clustered the first six items of the questionnaire (Q1–Q6) by averaging their values because they are all part of the main factor embodiment (Longo et al., 2008; Tajadura-Jiménez et al., 2012b; Romano et al., 2021; Tosi and Romano, 2022). We also clustered the remaining ten items of the questionnaire (Q7–Q16) by averaging their values because they belong to the original enfacement questionnaire (Tajadura-Jiménez et al., 2012b). To examine the subjective experience of embodiment elicited by the different stimulations (visuotactile vs. visuomotor vs. exposition), we ran a repeated measures analysis of variance (rmANOVA) with a within-subject design that covered a 2 (Congruency) × 3 (Stimulation) × 2 (Questionnaire) full-factorial model. The factor named Questionnaire controlled whether there were any differences between the embodiment and the enfacement constructs, as assessed by the respective items. Significant effects have been interpreted by inspecting 95% Confidence Intervals. The analyses investigating the different subcomponents of the embodiment sensation (i.e., Ownership–Q1-Q2; Agency–Q3-Q4; Location–Q5-Q6) are included in the Supplementary material. We ran a rmANOVA with a within-subject design that covered a 2 (Congruency) * 3 (Stimulation) model. We conducted two additional analyses to control for specific aspects of the experimental design. In the visuomotor condition, half of the participants saw the nodding movement, the other half saw the shaking one. To control for any influence of the type of movement presented, we ran an rmANOVA with a mixed within-/between-subject design that covered a 2 (Congruency) × 2 (Questionnaire) × 2 (Type of movement) factorial model. The results of the analysis are reported in the Supplementary materials. As for the enfacement questionnaire, we specifically looked at item Q10 to assess whether the experimental design influenced the similarity participants perceived with the avatar. The results of the 2 (Congruency) * 3 (Stimulation) rmANOVA are reported in the Supplementary materials. We conducted all the analyses with the ezANOVA function for the statistical software R (R Core Team, 2017).

## 3. Results

**FIGURE 3:**
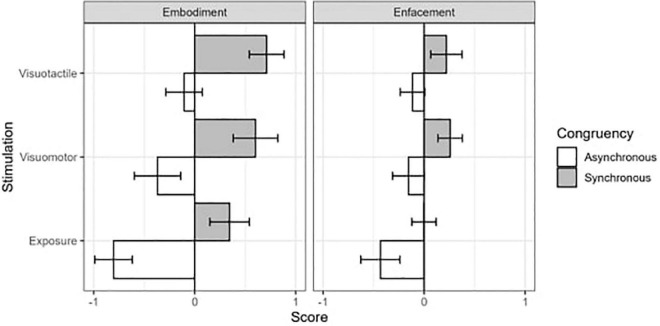
Results of the within-subjects 2 (Congruency) *3 (Stimulation) *2 (Questionnaire) repeated measure ANOVA on the averaged ipsatized answers to questionnaire statements. Gray and white columns display, respectively synchronous and asynchronous conditions. Error bars display confidence intervals.

We found significant main effects of Congruency [*F*(1,22) = 82.28, *p* ≤ 0.001, η^2^_*G*_ = 0.40] and Stimulation [*F*(2,44) = 10.93, *p* = 0.001, η^2^_*G*_ = 0.14] (Figure 3). These results revealed greater embodiment values in the synchronous condition (CI: 0.16; 0.55) than in the asynchronous one (−0.53; −0.13). Moreover, participants showed higher embodiment sensation after the visuotactile (CI: −0.03; 0.39) and visuomotor (CI: −0.16; 0.32) stimulations as compared to the exposure condition (CI: −0.47; 0.03). We also found a significant interaction between Congruency and Questionnaire [*F*(1,22) = 118.76, *p* ≤ 0.001, η^2^_*G*_ = 0.11], suggesting that the embodiment statements caught a greater difference between the synchronous and asynchronous stimulations as compared to the enfacement items (Figure 3). Moreover, the interaction between Stimulation and Questionnaire resulted to be significant [*F*(2,44) = 1.59, *p* ≤ 0.05, η^2^_G_ = 0.02], showing greater embodiment ratings as compared to the enfacement one only after the visuotactile stimulation (Figure 3).

No further significant effects emerged (all other *p*-values > 0.15). The results obtained from each specific subcomponent of the embodiment construct and from the additional control analyses are reported in Supplementary Tables 1–3.

## 4. Discussion

The present study aimed to verify the possibility of eliciting enfacement over computer generated faces and test which stimulation condition elicits the stronger illusion. We created CG faces with the software Character Creator, a new generation software allowing the creation of high-quality texture 3-D objects and avatars starting from face photographs. Although other studies used avatars in embodiment paradigms, we aimed to systematically investigate different enfacement stimulation conditions on those types of stimuli. The ultimate goal was to set a methodological reference to apply enfacement to face processing research. The role of visuotactile stimulation in enfacement paradigms has already been extensively studied. However, a visuomotor stimulation, not requiring an acquisition system for online tracking, has been investigated less frequently. Moreover the role of mere exposure, to the best of our knowledge, has never been studied. In our study, the enfacement illusion was compared among visuotactile stimulation, visuomotor stimulation, and exposure condition. Each stimulation comprised a congruent (i.e., synchronous) and an incongruent (i.e., asynchronous) condition. Our results suggest a difference in congruency (synchronous vs. asynchronous) where congruent stimulation elicited higher enfacement effects than incongruent. Our results confirm that a multisensory stimulation, either visuotactile or visuomotor, administered with spatial and temporal congruency is able to elicit enfacement. As for the visuomotor stimulation, it is important to underline that we used a guided movement as visuomotor stimulation. As already stated, the advantage of active over non-active movement to elicit embodiment is not clear in the current literature (Dummer et al., 2009; Kalckert and Ehrsson, 2014). Our results confirm that even a guided movement as a visuomotor stimulation is able to elicit enfacement. The method we used has the advantage of being more accessible and feasible with respect to active visuomotor stimulation because it does not require any facial motion capture system or complete immersive virtual reality environment. This result is in line with previous studies about embodiment and enfacement illusions, where the synchronous condition elicits a stronger illusion effect as compared to the asynchronous one (Longo et al., 2008; Tsakiris, 2008; Kilteni et al., 2015; Porciello et al., 2018).

Crucially, our results indicate that even the mere exposure to a CG face elicits a stronger enfacement effect when the face is presented in a congruent position as compared with a reversed face.

Aside from investigating the role of congruency, we also directly assessed whether there was any difference between the different stimulations we used (i.e., visuotactile, visuomotor, and exposure). Results reveal that visuotactile and visuomotor stimulation conditions create a stronger illusion over the virtual face as compared to the simple exposure condition. Even if the mere exposure is enough to induce an embodiment effect, in line with La Rocca et al. (2020), the effect is significantly weaker as compared to multisensory stimulation.

Moreover, we observed a significant interaction between congruency and the used questionnaire. This result indicates that the adapted version of the embodiment questionnaire is more sensitive in capturing the difference between synchronous and asynchronous stimulation as compared to the enfacement questionnaire. Thus, it appears clear that methodological research on the enfacement questionnaires is still needed. For example, recent literature uses a self-recognition task on a continuum of morphed images ranging between two identities. This serves to investigate the level of enfacement with the seen avatars. In fact, this measure should implicitly tell us what is the identification of the participant with a different identity (Deltort et al., 2022). It would be interesting to use it in future studies as it is an implicit measure which could be best to avoid test-retest effects. This measure would be helpful to investigate enfacement also in clinical populations (Ferroni et al., 2019; Deltort et al., 2022).

A possible limitation of the present study is that we created for each gender two avatars that we assigned to participants. However, we did not control for the similarity of appearance of our avatars with the participants. As a matter of fact, Fribourg et al. (2020) find that avatar appearance impacts the sense of embodiment less than other dimensions, such as control over it and its point of view (Fribourg et al., 2020). The authors suggest that this result may depend on the task used. On the other hand, Waltemate et al. (2018) find that personalized avatars significantly increase body ownership and sense of presence.

To explore its potential role, we looked at Q10 from our questionnaire (i.e., “It seemed like the other’s face began to resemble my own face”). This result is described in Supplementary Table 3 and suggests that in the synchronous condition, participants perceive the avatar as more similar to themselves than in the asynchronous condition. This result is interesting though only exploratory. Moreover, we cannot be sure whether the similarity between the participant and the avatar caused the embodiment effect or whether the perceived similarity was induced by the experimental manipulation. Thus, future research should quantitatively investigate the relationship between the similarity of the avatar to the participant and the enfacement effect. A further limitation of the present study regards the visuomotor stimulation condition. Two movements were used between participants: head nodding and shaking. We decided to use them both to avoid our results being driven by the potential valence of movements. In a control analysis, we checked whether there was a difference in enfacement scores depending on the presented movement. We expected the movements to equally elicit enfacement: however, we found higher scores in the group presented with the shaking movement. Nevertheless, this difference did not alter the main results of the present study. This analysis is reported in Supplementary Table 2. This result could be due to a difference in the foveal representation of the observed faces during stimulation, with the shaking face being easier to keep under fixation. However, this hypothesis is only speculative as we do not have enough data to drive conclusions. This result suggests that future studies should be careful in choosing the specific movement for visuomotor stimulation.

In conclusion, computer-generated faces can be a valid alternative to real faces to elicit enfacement. Moreover, their suitability is proved even for visuotactile and visuomotor stimulation conditions. From a procedure point of view, the novelty of the study is that it verifies enfacement illusion even in a setting of augmented reality. This makes it possible for other researchers not to use a VR headset or a completely immersive procedure but just a computer screen. Being able to embody a face of an avatar opens a wide range of possibilities in face processing research. In fact, the software used in the present experiment allows the manipulation of characteristics of the avatar’s face in virtual reality environments such as facial features appearance (i.e., different configurations of features and sizes), facial expressions, ethnicity, gender spectrum, and age span.

## Data availability statement

The datasets presented in this study can be found in online repositories. The names of the repository/repositories and accession number(s) can be found below: https://osf.io/cf8qv/?view_only=efd1bb4b124a4c12b295c5f31ea8bc20.

## Ethics statement

The studies involving human participants were reviewed and approved by the Ethical Committee of the University of Milano–Bicocca protocol number: RM-2021-392. The patients/participants provided their written informed consent to participate in this study.

## Author contributions

SL and SG provided the initial conception, organization, and main writing of the text. GT analyzed the data, prepared all figures and tables, and contributed to the writing of the text. EF collected the data. RD supervised the study and read and approved the draft. All authors contributed to the article and approved the submitted version.

## Footnotes

1
https://www.reallusion.com/character-creator/
