# Supplementary material for: Look at me now! Enfacement illusion over computer-generated faces
Source: Front Hum Neurosci. 2023 Mar 9;17:1026196. doi: 10.3389/fnhum.2023.1026196 (PMC10034087; doi:10.3389/fnhum.2023.1026196)
Supplement: Supplementary file 1 [file Data_Sheet_1.docx]

Supplementary Material

# Supplementary Data

In the supplementary material section, we reported additional results that go more into the details of the embodiment questionnaire decomposition and control for specific aspects of the experimental design.

**Embodiment additional results**

Looking at the three sub-components of the embodiment sensation, we always found significant effects of congruency, confirming higher values of ownership, agency and location in the synchronous condition than in the asynchronous one. We also found significant effects of Stimulation for the three subcomponents with lower values in the exposure condition.

We also found a significant interaction for the location component showing higher differences between the synchronous and asynchronous conditions during the exposure phase as compared to the others. *(Table SM1)*

**Visuomotor additional results**

Looking at the visuomotor stimulation, we confirmed the significant main effect of congruency and the significant interaction between congruency and questionnaire. Additionally, we found a significant main effect of the type of movement presented to the participants. In particular, the group who saw the shaking movement showed higher scores (CI: 0.02; 0.48) as compared to the group who saw the nodding movement (CI: -0,30; -0.17). *(Table SM2)*

**Enfacement additional results**

Looking at the similarity item of the Enfacement questionnaire, we found a significant effect of congruency. This result shows higher values of perceived similarity in the synchronous condition than in the asynchronous one. *(Table SM3)*

# Supplementary Tables

|  | **df** | | **f** | **p** | **df** | **f** | **p** | **df** | **f** | **p** |
| --- | --- | --- | --- | --- | --- | --- | --- | --- | --- | --- |
|  | ***Ownership*** | | | | ***Agency*** | | | ***Location*** | | |
| **Congruency** | 1 | | 36.68 | < .001*** | 1 | 64.02 | < .001*** | 1 | 85.93 | < .001*** |
| **Stimulation** | 2 | | 2.67 | 0.07 | 2 | 3.08 | < .05* | 2 | 26.84 | < .001*** |
| **Congruency* Stimulation** | 2 | | 0.19 | 0.8 | 2 | 1.11 | 0.33 | 2 | 4.89 | < .01** |
| **Residuals** |  | 132 |  |  | 132 |  |  | 132 |  |  |

*Table SM1*. Results of the 2 (Congruency) * 3 (Stimulation) repeated measures analysis of variance for each factor of the embodiment questionnaire.

|  | **df** | **f** | **p** |
| --- | --- | --- | --- |
| **Congruency** | 1 | 58.72 | < .001*** |
| **Questionnaire** | 1 | 0.52 | 0.473 |
| **Type of movement** | 1 | 12.16 | < .001*** |
| **Congruency* Type of movement** | 1 | 1.95 | 0.167 |
| **Congruency* Questionnaire** | 1 | 9.55 | < .01** |
| **Type of movement * Questionnaire** | 1 | 0.20 | 0.653 |
| **Congruency* Type of movement * Questionnaire** | 1 | 0.75 | 0.388 |
| **Residuals** | 84 |  |  |

*Table SM2*. Results of the 2 (Congruency) x 2 (Questionnaire) x 2 (Type of movement) repeated measures analysis of variance on the averaged ipsatized answers to questionnaire statements.

|  | **df** | | **f** | **p** |
| --- | --- | --- | --- | --- |
|  | ***Ownership*** | | | |
| **Congruency** | 1 | | 7.71 | < .01** |
| **Stimulation** | 2 | | 1.56 | 0.21 |
| **Congruency* Stimulation** | 2 | | 0.66 | 0.52 |
| **Residuals** |  | 132 |  |  |

*Table SM3*. Results of the 2 (Congruency) * 3 (Stimulation) repeated measures analysis of variance for the similarity item (Q10).
